# William McKinley

**Published:** 1901-10

**Authors:** 


### EDITORIALS.

### WILLIAM McKINLEY.

THE nation has been called upon to mourn the death of the President of the United States. Again we realize, after nineteen hundred years, how one man who has passed his life in the service and for the good of those around him can be sacrificed by the evil of one of those for whom he had labored to do good. The country at large and the seventy-five millions of people as individuals mourn the death of the President and feel acutely the disgrace brought upon them by the dastardly murderer, a citizen of this free country.

It is a glorious tribute to the free institutions of the United States to have seen how sectional feeling and all the animadversions of various political parties have been lost in this national horror, and each and every man has been drawn in silent sympathy to his neighbor.

But the death of President McKinley brings scarcely a shadow of disaster to the country. Representing a great people, he had so welded and riveted the principles long ago forged by Washington, Jefferson and Lincoln that his passing away becomes only that of a man. It is as a man that we as individuals mourn Mr. McKinley.

A prominent Senator says: "From the moment he took his place in the White House President McKinley grew in the estimation, the friendship, and the love of the American people. His virtues and his statesmanship will be the greater appreciated as time rolls on and his private life and public acts shall become the more and the better known."

Personally, he had the most charming manners of any gentleman in the United States. Man, woman, or child who ever had the honor of an interview with him left him with a feeling of content. The man realized that Mr. McKinley was a master of every detail in which he was consulted; the woman felt that he was a man of sympathy; the child left him imbued with the ambition to be a great man.

HUIDEKOPER.

